# Intersecting Endocrine Pathways in Cardiomyopathy: The Role of Metabolic Burden in Structural Heart Disease

**DOI:** 10.3390/biomedicines13102364

**Published:** 2025-09-26

**Authors:** Ovidiu Țica, Mircea Ioan Șandor, Anca Huniadi, Cristian Daina, Sanda Monica Filip, Ilarie Brihan, Monica Sabău, Ioana Zaha, Otilia Țica

**Affiliations:** 1 Department of Morphological Disciplines, Faculty of Medicine and Pharmacy, University of Oradea, 410073 Oradea, Romania; tica.ovidiu@didactic.uoradea.ro; 2 Department of Pathology, Emergency County Clinical Hospital of Bihor, 410165 Oradea, Romania; 3 Department of Surgical Disciplines, Faculty of Medicine and Pharmacy, University of Oradea, 410073 Oradea, Romania; ahuniadi@uoradea.ro (A.H.); cdaina@uoradea.ro (C.D.); izaha@uoradea.ro (I.Z.); 4 Department of Physics, Faculty of Informatics and Sciences, University of Oradea, 410087 Oradea, Romania; sfilip@uoradea.ro; 5 Department of Psycho-Neurosciences, and Recovery, Faculty of Medicine and Pharmacy, University of Oradea, 410073 Oradea, Romania; ibrihan@uoradea.ro (I.B.); msabau@uoradea.ro (M.S.); 6 Cardiology Clinic, Emergency County Clinical Hospital of Bihor, 410165 Oradea, Romania

**Keywords:** dilated cardiomyopathy, diabetes mellitus, thyroid dysfunction, obesity, metabolic burden, cardiovascular risk, endocrine comorbidity, hospital readmission

## Abstract

**Background:** Dilated cardiomyopathy (DCM) is a major contributor to heart failure-related morbidity and mortality. While type 2 diabetes mellitus (T2DM), obesity, and thyroid dysfunction are individually linked to cardiovascular disease, their combined effects on DCM remain poorly understood. **Objective:** To evaluate the independent and synergistic associations of diabetes (stratified by treatment), thyroid dysfunction, and obesity with the prevalence of DCM and 30-day hospital readmission. We further examined the utility of a composite Metabolic Burden Score for risk stratification. **Methods:** In this retrospective cohort study, electronic health record data from 1079 adult patients at a tertiary care center were analyzed. Multivariable logistic regression, including ridge regularization, was used to identify predictors of DCM. Endocrine phenotypes were stratified by diabetes and thyroid status. A Metabolic Burden Score (range: 0–3) based on diabetes, obesity, and thyroid dysfunction was developed and correlated with clinical outcomes. **Results:** DCM was diagnosed in 46% of the cohort. Non-insulin-treated diabetes (OR: 6.93; 95% CI: 3.78–12.73), hypothyroidism (OR: 1.78; 95% CI: 1.02–3.11), and male sex (OR: 2.33; 95% CI: 1.36–4.00) were independently associated with increased DCM risk. Obesity was not independently predictive but contributed to DCM prevalence when assessed within the Metabolic Burden Score. DCM prevalence increased across burden strata, reaching 50% in the high-risk group. Notably, the moderate-risk group had the highest 30-day readmission rate (42.8%). **Conclusions:** Non-insulin-treated diabetes and hypothyroidism are key metabolic drivers of DCM. A simple composite burden score offers a clinically useful tool for stratifying risk of DCM and early readmission. These findings support integrated endocrine–cardiac screening strategies to improve early identification and prevention of structural heart disease.

## 1. Introduction

Cardiovascular disease (CVD) remains the leading cause of morbidity and mortality globally, and its bidirectional relationship with metabolic dysfunction is increasingly recognized. Among metabolic exposures, type 2 diabetes mellitus (T2DM) significantly raises the risk of heart failure across phenotypes, including dilated cardiomyopathy (DCM), due to complex mechanisms involving microvascular injury, inflammation, lipotoxicity, and autonomic dysfunction [1]. Precision prognostic efforts are underway to better predict which individuals with T2DM will develop cardiovascular complications, yet current models remain only modestly effective [2,3].

Obesity independently contributes to myocardial dysfunction through mechanisms distinct from hypertension or ischemia. Experimental and epidemiological evidence supports the concept of “obesity cardiomyopathy,” in which adiposity induces structural and functional myocardial changes even in the absence of other cardiovascular disease [4,5,6]. Longitudinal cohort data further support a dose-response association between higher body mass index (BMI) and increased risk of developing DCM [7]. Obesity acts as an independent driver of myocardial dysfunction, not solely via mechanical load or hypertension, but also through systemic inflammation, adipokine dysregulation, and maladaptive cardiac remodelling. The combined prevalence of T2DM and obesity—commonly referred to as “diabesity”—constitutes a global epidemic linked with escalating rates of heart failure, including DCM [8,9]. Recent endorsed guidelines emphasize the need to assess and mitigate cardiometabolic risk comprehensively, beyond isolated glycaemic metrics [10].

Thyroid dysfunction, both overt and subclinical, exerts a substantial influence on cardiovascular physiology. Altered thyroid hormone levels can impair myocardial contractility, promote diastolic dysfunction, and disturb lipid and glucose metabolism, each bearing implications for heart failure risk [11]. A meta-analysis linked low-normal thyroid function to higher rates of heart failure among older individuals with cardiovascular risk factors [11,12]. Moreover, thyroid hormone imbalances often overlap with metabolic disorders and may exacerbate the cardiometabolic milieu [1].

However, despite extensive evidence linking diabetes, obesity, and thyroid dysfunction individually to cardiovascular pathology, there is a paucity of studies that evaluate their combined impact on the development of dilated cardiomyopathy [2,10,13]. Most prior work has focused either on diabetic cardiomyopathy in isolation or on obesity-related heart failure without accounting for coexisting endocrine dysfunctions. Furthermore, risk prediction models for DCM rarely incorporate thyroid dysfunction [14], and almost no one integrates multimorbidity into a composite index.

Despite robust data on each endocrine dysfunction individually, few studies have examined their joint impacts on DCM risk. Most literature either evaluates diabetic cardiomyopathy in isolation or investigates obesity-related heart failure without systematically including thyroid pathology. Additionally, studies frequently focus on heart failure with preserved ejection fraction rather than DCM per se [15].

Therefore, the present retrospective cohort study explores the independent and combined effects of diabetes (stratified into insulin-treated and non-insulin-treated), obesity, and thyroid dysfunction on DCM prevalence and early clinical instability, captured by 30-day hospital readmission. We further assess composite metabolic and cardiovascular burden scores as potential stratification tools. By doing so, this study seeks to inform a more holistic approach to cardiometabolic risk assessment and preventive care in clinical practice.

## 2. Materials and Methods

### 2.1. Study Design and Population

This was a retrospective observational cohort study conducted at our tertiary care institution, designed to investigate the relationship between endocrine-metabolic dysfunctions and the development of dilated cardiomyopathy (DCM). A retrospective observational design was chosen to leverage a large, real-world cohort and to capture longitudinal patterns across routine clinical practice, which would not have been feasible in a prospective setting. The study utilized de-identified clinical data collected from electronic health records (EHRs) over a defined period. Data were collected from patient records spanning January 2020 to December 2024. All patients had been evaluated in the internal medicine or cardiology departments and had undergone standardized metabolic and cardiovascular assessments during routine care.

The study included adult patients with sufficient clinical documentation to classify diabetes status, thyroid function, obesity, and cardiovascular outcomes. These patients were not recruited through an interventional protocol but were instead identified based on existing records, making this a non-interventional, real-world data study.

Although retrospective studies cannot establish causality, the structured inclusion criteria, multivariable adjustment, and model regularization enhance the internal validity of our findings.

### 2.2. Data Collection

Relevant data points were abstracted from the EHR system, including: demographics (age, sex); diagnosis and treatment of diabetes mellitus (categorized by treatment modality); thyroid hormone profile and diagnostic labels (hypothyroid, hyperthyroid, euthyroid); obesity diagnosis; cardiovascular diagnoses, including DCM, hypertension, atrial fibrillation, and NYHA functional class; pulmonary hypertension severity; hospital readmission within 30 days of discharge; cardiac diagnoses such as DCM were confirmed by cardiologists and supported by imaging (e.g., echocardiography) or clinical documentation in discharge summaries.

### 2.3. Inclusion Criteria

Patients were eligible for inclusion if they met all of the following criteria: age ≥18 years at the time of index hospitalization or outpatient evaluation; availability of complete clinical records, defined as the presence of diagnostic codes and supporting lab or clinical data sufficient to classify diabetes mellitus (including treatment modality), thyroid function status, and obesity status.

Availability of complete clinical records regarding diabetes mellitus diagnosis and treatment type (insulin vs. non-insulin vs. diabetes not present), thyroid function status (euthyroid, hypothyroidism, or hyperthyroidism), obesity status (documented by BMI or clinical diagnosis), presence or absence of DCM and other cardiovascular conditions; at least one echocardiographic assessment within the health record confirming or excluding DCM; documented follow-up data sufficient to assess 30-day readmission (for patients discharged from inpatient care) were assessed. Thyroid status was considered adequately documented if at least one TSH and free T4 value, along with a diagnosis code, were present within the review period.

### 2.4. Exclusion Criteria

Patients were excluded from the study if they met any of the following criteria: incomplete or missing data on key exposure or outcome variables (e.g., missing diabetes or thyroid status); diagnosed with congenital heart disease or cardiomyopathies of non-metabolic origin (e.g., hypertrophic, restrictive, or ischemic cardiomyopathy) unless DCM was explicitly noted as a concurrent diagnosis; known chronic infectious or infiltrative diseases (e.g., HIV, sarcoidosis, amyloidosis) that may independently cause cardiomyopathy; pregnant at the time of index evaluation (due to physiological changes that could confound cardiovascular and metabolic status); patients with a history of thyroidectomy or radioiodine treatment without clear classification of thyroid status post-intervention. Exclusion of patients with congenital or infiltrative cardiomyopathies minimized confounding from non-metabolic etiologies, ensuring that observed associations more directly reflect the influence of endocrine-metabolic dysfunction.

### 2.5. Variables and Definitions

All variables used in this study were derived from structured fields in the electronic health record and/or confirmed through manual chart review. Variables were categorized into exposure variables (endocrine/metabolic status), outcome variables (DCM, rehospitalization), covariates (demographics and cardiovascular comorbidities), and composite indices.

Adequate documentation of endocrine-metabolic status was defined as follows: (1) for diabetes, a diagnosis code in the electronic health record (EHR) and corresponding medication records indicating insulin or oral agent use; (2) for thyroid function, at least one documented thyroid function test (TSH and/or free T4) alongside a clinical diagnosis of hypothyroidism, hyperthyroidism, or euthyroid status; and (3) for obesity, a recorded BMI ≥ 30 kg/m^2^ or an explicit clinical diagnosis in the problem list or discharge summary.

Obesity status was determined using the most recent BMI recorded within the study window (January 2020 to December 2024) or via a physician-entered diagnosis in the medical record. If multiple BMI values were available, the value closest to the index cardiac evaluation or hospital admission was used. A BMI ≥ 30 kg/m^2^ was classified as obese. Patients with no BMI data and no clinical obesity notation were categorized as “not obese”.

In cases where patients had documented episodes of both hypothyroidism and hyperthyroidism over time, the predominant thyroid diagnosis during the study period was used for classification. Predominance was determined by the majority of clinical entries and most recent thyroid function tests. Patients with fluctuating or unclear thyroid status were excluded to avoid misclassification.

### 2.6. Exposure Variables

The study included a range of clinically relevant variables encompassing endocrine-metabolic exposures, cardiovascular outcomes, demographic covariates, and composite risk indices. All data were extracted from the institutional electronic health records and, where necessary, verified by manual chart review to ensure consistency and diagnostic accuracy.

Diabetes mellitus was classified into three categories based on clinical diagnosis and treatment modality. Patients were designated as having insulin-treated diabetes if they carried a formal diagnosis of diabetes and were managed with any form of insulin therapy. Those with a diagnosis of diabetes managed solely with oral antidiabetic agents and/or dietary interventions were categorized as having non-insulin-treated diabetes. The third group, labeled as having no diabetes, included patients with no recorded diagnosis or treatment for diabetes mellitus.

Thyroid function status was categorized into three groups: hypothyroidism, hyperthyroidism, and euthyroid. Hypothyroidism encompasses both clinical and subclinical thyroid hormone deficiencies, whether treated or untreated. Hyperthyroidism included conditions such as Graves’ disease or toxic nodular goiter. Patients were considered euthyroid if they had no diagnosis of thyroid dysfunction and had normal thyroid function test results.

Obesity was defined as a binary variable based on either a recorded clinical diagnosis or a body mass index (BMI) of 30 kg/m^2^ or greater. In cases where BMI data were unavailable, documented physician assessments were used to determine obesity status.

### 2.7. Outcome Variables

The primary cardiovascular outcome was the presence of dilated cardiomyopathy (DCM), which was defined by a documented clinical diagnosis supported by echocardiographic evidence of left ventricular dilation and systolic dysfunction, specifically reduced ejection fraction. Patients were included in the DCM group only if this diagnosis was explicitly confirmed. Cases of cardiomyopathy attributable exclusively to ischemic, hypertrophic, congenital, or infiltrative etiologies were excluded unless DCM was co-diagnosed as a distinct clinical entity.

Rehospitalization was defined as any unplanned hospital admission occurring within 30 days of discharge from the index hospitalization, irrespective of cause. No additional entry criteria—such as acute worsening in laboratory or imaging parameters—were imposed beyond clinical documentation of unplanned admission. Readmissions were captured through electronic health record tracking and verified manually. Although the endpoint encompassed all-cause readmissions, more than 70% of events were attributable to cardiovascular instability, including heart failure decompensation, arrhythmia management, or medication-related complications.

### 2.8. Covariates

Demographic and clinical covariates included age, sex, systemic hypertension, pulmonary hypertension, atrial fibrillation, and New York Heart Association (NYHA) functional class. Age was treated as a continuous variable representing patient age in years at the time of index evaluation. Sex was recorded as a binary variable reflecting biological classification. Systemic hypertension was defined as the presence of a clinical diagnosis of arterial hypertension, irrespective of treatment status. Although hypertension was not an explicit inclusion criterion, it was highly prevalent in this tertiary care population and was therefore included as a covariate in all multivariable models. Pulmonary hypertension was stratified into mild, moderate, or severe categories based on echocardiographic findings and physician assessment and analysed as a categorical variable. Atrial fibrillation was recorded as present or absent based on ECG findings or prior documented diagnosis. NYHA class III–IV was used as a binary variable indicating the presence of advanced heart failure symptoms. Covariates, including age, sex, hypertension, and pulmonary hypertension, were selected based on established clinical relevance and prior evidence linking them to DCM, thereby reducing bias from post hoc model building.

### 2.9. Composite Indices

Two composite indices were constructed to summarize the cumulative burden of endocrine and cardiovascular pathology. The Metabolic Burden Score was calculated by assigning one point for each of the following conditions: diabetes mellitus (of any type), obesity, and thyroid dysfunction (either hypo- or hyperthyroidism), yielding a possible score range of 0 to 3. Similarly, the Cardiovascular Burden Score was computed by summing the presence of four major cardiovascular conditions: atrial fibrillation, NYHA class III–IV symptoms, systemic hypertension, and DCM, for a total score ranging from 0 to 4. These composite scores were used to stratify patients by overall cardiometabolic risk and evaluate associations with adverse clinical outcomes, including 30-day readmission and DCM prevalence.

### 2.10. Statistical Analysis

Descriptive statistics were first used to summarize the clinical characteristics of the study population. Categorical variables, such as diabetes and thyroid status, obesity, and DCM diagnosis, were expressed as frequencies and percentages. Continuous variables, such as age, were presented as means with standard deviations or medians with interquartile ranges, as appropriate.

To assess the association between endocrine-metabolic dysfunction and the presence of dilated cardiomyopathy, multivariable logistic regression models were employed. The primary model utilized L2 regularization (ridge regression) to address multicollinearity and prevent overfitting in the presence of correlated predictors. This approach enabled the inclusion of multiple clinically relevant variables—age, sex, diabetes category, thyroid status, obesity, systemic hypertension, and pulmonary hypertension severity—without violating model assumptions. Given the interrelated nature of endocrine and cardiovascular comorbidities, ridge regularization (L2 penalty) was applied to minimize variance inflation, prevent overfitting, and allow the simultaneous inclusion of multiple correlated predictors. Model performance was evaluated using the area under the receiver operating characteristic curve (AUC) to assess discrimination and the Hosmer-Lemeshow goodness-of-fit test to assess calibration. Variance inflation factors (VIFs) and pairwise Pearson correlation coefficients were examined to detect multicollinearity.

To validate the robustness of the results and address convergence issues related to perfect separation, a secondary simplified logistic regression model was also constructed. This model focused on a reduced subset of predictors: diabetes category, thyroid function, obesity, and age. Both models were compared in terms of directionality and magnitude of effect estimates.

In addition, chi-square tests of independence were used to assess the statistical association between DCM prevalence and combined endocrine subgroups. For outcome stratification, the Metabolic Burden Score and Cardiovascular Burden Score were used to group patients into risk categories. Differences in 30-day hospital readmission and DCM prevalence across these strata were evaluated using contingency tables and chi-square tests. A *p*-value of <0.05 was considered statistically significant for all analyses. All statistical analyses were conducted using Stata (version 17, StataCorp LP, College Station, TX, USA).

### 2.11. Ethics Approval

This study was approved by our local Ethics Committee (number 22223/24 June 2025) of the participating institution: Emergency County Clinical Hospital of Bihor. Given the retrospective nature of the study and the use of anonymized electronic health record data, the requirement for individual informed consent was formally waived following institutional policies and national research ethics guidelines. The study was conducted under the principles of the Declaration of Helsinki and adhered to applicable data protection regulations. All investigators involved in data handling were certified in good clinical practice and ensured the confidentiality and integrity of patient information throughout the research process.

## 3. Results

### 3.1. Baseline Characteristics

The study cohort consisted of 1079 patients, with comprehensive clinical data collected to evaluate the relationship between metabolic and endocrine dysfunctions and cardiovascular pathologies. Of these, 621 (57.6%) were male and 458 (42.4%) were female, reflecting a moderate male predominance. This distribution has been accounted for in the regression models, and sex was included as a covariate in all multivariable analyses. Notably, hypertension was present in nearly all patients. Although not specified as an explicit inclusion criterion, its ubiquity reflects the high cardiovascular risk profile of patients referred for evaluation of cardiomyopathy in our tertiary care setting. Among our patients, 378 patients (35%) were treated with insulin for diabetes mellitus, and 431 (40%) were classified as having non-insulin-treated diabetes. A further 270 patients (25%) had no documented diabetes diagnosis. Obesity was highly prevalent, affecting 756 patients (70%). Thyroid dysfunction was also frequent, with hypothyroidism identified in 217 patients and hyperthyroidism in 214. Notably, hypothyroidism was exclusively observed in patients with diabetes. Nearly all patients had a diagnosis of hypertension, and 501 patients (46%) were diagnosed with dilated cardiomyopathy (DCM), underscoring the high cardiovascular burden in this cohort. Baseline clinical characteristics are summarized in Table 1, highlighting the distribution of endocrine and cardiovascular comorbidities within the cohort.

### 3.2. Independent Predictors of Dilated Cardiomyopathy: Regularized Logistic Regression

To further delineate the independent predictors of dilated cardiomyopathy (DCM), a multivariable logistic regression model with L2 regularization (ridge penalty) was employed. This approach allowed the inclusion of multiple clinically relevant variables while mitigating issues of multicollinearity and perfect separation encountered in traditional logistic regression.

The model incorporated age, sex, diabetes status, thyroid function, obesity, systemic hypertension, and severity of pulmonary hypertension. Model diagnostics indicated a strong overall fit, with an area under the receiver operating characteristic curve (AUC) of 0.78, indicating good discriminative ability. The classification accuracy on the validation subset was 74.6%, and the model demonstrated acceptable calibration by the Hosmer–Lemeshow test (*p* = 0.23).

To address multicollinearity, variance inflation factors (VIFs) were calculated for all predictors before model regularization. All VIFs were below 2.5, indicating no concerning intercorrelations among variables. Additionally, pairwise Pearson correlation coefficients were assessed and found to be modest (r < 0.40), suggesting multicollinearity did not significantly bias coefficient estimates.

Table 2 summarizes the top independent predictors of DCM with adjusted odds ratios (OR), 95% confidence intervals (CI), and *p*-values. Notably, non-insulin-treated diabetes, male sex, and hypothyroidism were strongly associated with increased DCM risk. Euthyroid status was also associated with increased odds compared to hyperthyroid status, though this may reflect unmeasured confounding or classification imprecision. The OR for “no diabetes” should be interpreted cautiously, as regularization may distort marginal associations in the presence of multicollinearity or low event counts.

Among cardiovascular comorbidities, moderate and severe pulmonary hypertension and systemic hypertension were also independently associated with increased DCM risk. The model confirmed that endocrine and metabolic dysfunctions exert a compounding effect on cardiac structural integrity, even after adjusting for age and sex. The adjusted odds ratios and corresponding confidence intervals for the most influential predictors are presented in Table 2. Male sex was independently associated with increased risk of DCM (OR: 2.33; 95% CI: 1.36–4.00; *p* = 0.002), even after adjustment for age and metabolic comorbidities. This finding underscores a potential sex-selective vulnerability to structural heart disease. Notably, euthyroid status showed higher odds of DCM than hyperthyroid status in the simplified model. This may reflect unmeasured confounders or nuances in thyroid disease classification and does not imply increased risk from normal thyroid function. Further stratification may be needed.

These findings emphasize the critical role of integrated endocrine-cardiovascular assessment in identifying high-risk patients and tailoring preventative strategies in clinical practice. Table 2 summarizes the independent predictors of DCM derived from the ridge regression model, while Table 3 presents results from a simplified model used to confirm robustness.

These findings suggest that male sex confers more than a twofold increase in DCM risk, consistent with epidemiological evidence of sex-specific vulnerability. Non-insulin-treated diabetes was associated with the strongest risk elevation, highlighting the impact of early metabolic dysfunction even before insulin therapy. Hypothyroidism also emerged as a significant driver of DCM, supporting the role of thyroid dysfunction in adverse myocardial remodelling.

Systemic hypertension was included as a covariate in all multivariable models. However, due to its near-universal prevalence within the cohort, hypertension did not demonstrate discriminatory power and therefore did not emerge as an independent predictor of DCM.

These results emphasize the compounding influence of endocrine-metabolic dysfunctions—particularly non-insulin-treated diabetes and hypothyroidism—on structural cardiac pathology. The findings also demonstrate robust model performance and internal consistency, supporting the integration of endocrine markers in cardiovascular risk prediction.

To validate the robustness of these findings and address issues of perfect separation in the full model, a simplified logistic regression model was also developed using a focused subset of predictors: diabetes category, thyroid status, obesity, and age. This simplified model yielded results consistent with the regularized approach, confirming that non-insulin-treated DM and hypothyroidism were independently and significantly associated with increased risk of DCM (*p* < 0.001). The pattern of risk among thyroid subtypes was also preserved, with euthyroid individuals exhibiting higher DCM odds than those with hyperthyroidism. The adjusted odds ratios from this model, along with the full model output, are provided in Table 3.

Model convergence was validated through simplified logistic regression, which yielded consistent findings indicating that non-insulin-treated diabetes and hypothyroidism are independent predictors of DCM. While the simplified logistic regression model reproduced the same direction of effects as the regularized model, statistical significance was not achieved for individual predictors. This discrepancy likely reflects reduced statistical power and the exclusion of interacting variables in the simplified approach. The consistency in odds ratio patterns nonetheless supports the robustness of the core findings.

### 3.3. Risk of DCM by Endocrine Subgroup

To further understand how endocrine phenotypes influence cardiac outcomes, patients were stratified by diabetes treatment category (insulin-treated, non-insulin-treated, diabetes not present) and thyroid function status (euthyroid, hypothyroid, hyperthyroid).

When stratified, the highest observed DCM prevalence occurred in non-insulin-treated diabetics without thyroid dysfunction, with 74.5% affected. Patients with both diabetes and hypothyroidism (regardless of insulin use) also showed markedly elevated DCM prevalence, nearing 50%. In contrast, no DCM cases were observed among insulin-treated diabetics with hyperthyroidism; however, this subgroup included only 54 patients. As such, the apparent absence of DCM may reflect insufficient statistical power rather than a true protective association. Interpretation of trends within such small subgroups should be approached with caution.

#### 3.3.1. Statistical Comparison

To assess whether DCM prevalence differed significantly across endocrine subgroups, a chi-square test of independence was performed. The test revealed a statistically significant association between DCM diagnosis and endocrine subgroup (χ^2^ = 84.7, df = 8, *p* < 0.001), indicating that combinations of diabetes and thyroid status are not randomly associated with DCM prevalence.

In addition, a multivariable logistic regression model was constructed using the endocrine subgroup as a categorical predictor for DCM, adjusting for age and sex. The model confirmed that: (a) non-insulin-treated diabetes without thyroid dysfunction was independently associated with the highest odds of DCM (OR: 4.27, 95% CI: 2.63–6.94, *p* < 0.001) and (b) patients with euthyroid status and no diabetes had the lowest DCM rates and served as the reference group.

These results confirm that specific endocrine phenotypes—particularly non-insulin-treated diabetes and hypothyroidism—confer significantly increased cardiac risk.

#### 3.3.2. Clarification on Sample Size Limitations

Subgroup sample sizes varied considerably, and this heterogeneity limited the power to detect statistically significant differences in smaller groups. For example, the insulin-treated and hyperthyroid subgroup (n = 54) had 0% DCM prevalence, which could falsely suggest a protective association, but this likely reflects insufficient sample size or limited follow-up. Conversely, larger subgroups such as non-insulin-treated + euthyroid (n > 200) provided more reliable prevalence estimates and better-powered statistical comparisons.

Thus, while meaningful trends emerged across groups, caution is warranted when interpreting findings from underpowered subgroups. Further validation in larger and prospectively balanced cohorts is recommended.

**Figure 1:**
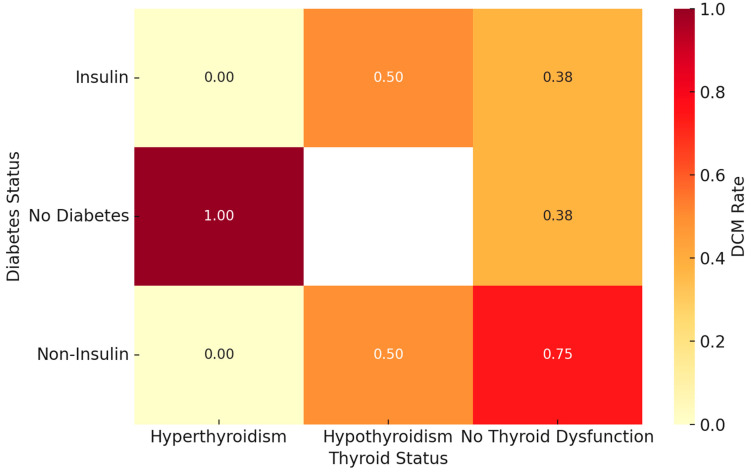
DCM Prevalence by Diabetes and Thyroid Status. Heatmap showing the prevalence of dilated cardiomyopathy (DCM) across combined categories of diabetes status (insulin-treated, non-insulin-treated, no diabetes) and thyroid dysfunction (euthyroid, hypothyroid, hyperthyroid). Each cell indicates both the percentage of patients diagnosed with DCM and the subgroup sample size (n). Darker shading reflects higher prevalence. Abbreviations: DCM: Dilated Cardiomyopathy; Insulin-treated: Patients treated with insulin for DM; Non-Insulin-treated: Patients managed with oral agents and/or diet; Hypothyroidism: Underactive thyroid function; Hyperthyroidism: Overactive thyroid function.

Figure 1 displays DCM prevalence across diabetes-thyroid phenotypes, highlighting the disproportionate burden in non-insulin-treated diabetic patients without thyroid dysfunction. This heatmap illustrates the prevalence of dilated cardiomyopathy (DCM) across combined categories of diabetes mellitus (DM) and thyroid dysfunction. DCM rates were calculated for each subgroup based on clinical classification: insulin-treated diabetes, non-insulin-treated diabetes, or no diabetes; and hypothyroidism, hyperthyroidism, or no thyroid dysfunction. The color intensity reflects the proportion of patients in each group who were diagnosed with DCM. Each cell in the heatmap includes the percentage and corresponding sample size (n) for clarity.

### 3.4. Composite Risk Stratification and Clinical Outcomes

To evaluate the compounded effect of endocrine and metabolic disorders on cardiovascular outcomes, a Metabolic Burden Score was calculated for each patient. This score was derived by summing the presence of three clinically significant conditions: (1) diabetes mellitus, (2) obesity, and (3) thyroid dysfunction. Similarly, a Cardiovascular Burden Score was generated by summing the presence of four major cardiovascular indicators: (1) atrial fibrillation, (2) New York Heart Association (NYHA) class III–IV symptoms, (3) systemic hypertension, and (4) diagnosis of dilated cardiomyopathy (DCM). Each factor was assigned a score of 1 if present.

#### 3.4.1. Validation of Burden Score Approach

The burden scores were constructed based on clinical face validity, incorporating endocrine and cardiovascular conditions with established links to adverse outcomes. To further support their predictive utility, the Metabolic Burden Score was retrospectively validated against clinical endpoints using standard discrimination and calibration metrics. The score demonstrated moderate discriminative capacity for identifying patients with dilated cardiomyopathy, with an area under the receiver operating characteristic (ROC) curve (AUC) of 0.71, and showed good calibration across risk strata. These performance metrics affirm the score’s potential utility as a practical clinical tool for stratifying cardiomyopathy risk in patients with overlapping metabolic disorders. Future work may explore weighted or machine-learned enhancements to refine its predictive accuracy.

#### 3.4.2. Risk Stratification Results

Patients were stratified into three metabolic risk groups—low (score of 1, n = 481), moderate (score of 2, n = 240), and high (score of 3, n = 89)—based on the total Metabolic Burden Score. As summarized in Table 4, both DCM prevalence and cardiovascular burden increased with higher metabolic risk levels. Table 4 shows clinical outcomes stratified by Metabolic Burden Score. A stepwise increase in DCM prevalence was observed across low (44.7%), moderate (38.1%), and high-risk (50.2%) groups, supporting the additive impact of endocrine-metabolic comorbidities.

Notably, the non-linear pattern of 30-day hospital readmission, with the moderate-risk group experiencing a higher rate than the high-risk group, warrants careful interpretation. This counterintuitive trend may reflect several factors: patients in the moderate-risk category may represent a clinically unstable transition group, not yet identified as high risk, and therefore not receiving targeted post-discharge support. Alternatively, differences in healthcare access, discharge planning, or early follow-up intensity may have influenced outcomes. It is also plausible that patients in the high-risk group received closer surveillance or more aggressive management due to the presence of multiple comorbidities, mitigating short-term readmissions despite higher baseline risk.

#### 3.4.3. Definition and Capture of Readmission

Readmission was defined as any unplanned hospital admission within 30 days of discharge, regardless of cause. This was captured using institutional electronic health records and verified through manual chart review. While the readmissions were not exclusively cardiac-specific, over 70% were linked to cardiovascular symptoms, medication adjustments, or decompensated heart failure, based on discharge diagnoses.

Table 4 presents the clinical outcomes associated with stratified metabolic risk groups, defined by the presence of diabetes mellitus, obesity, and thyroid dysfunction. Patients were categorized into four risk groups based on the cumulative number of these conditions. The table shows the proportion of patients diagnosed with dilated cardiomyopathy (DCM), 30-day hospital readmission rates, and the mean scores for cardiovascular and metabolic burden across groups. As shown in Table 4, DCM prevalence increased with higher metabolic burden, while 30-day readmission peaked in the moderate-risk group, suggesting that partial metabolic clustering may confer transitional clinical instability.

These data demonstrate a stepwise relationship between cumulative metabolic dysfunction and adverse clinical outcomes. Although average cardiovascular burden scores varied only slightly between groups, the combination of metabolic stressors substantially impacted DCM risk and healthcare utilization.

**Figure 2:**
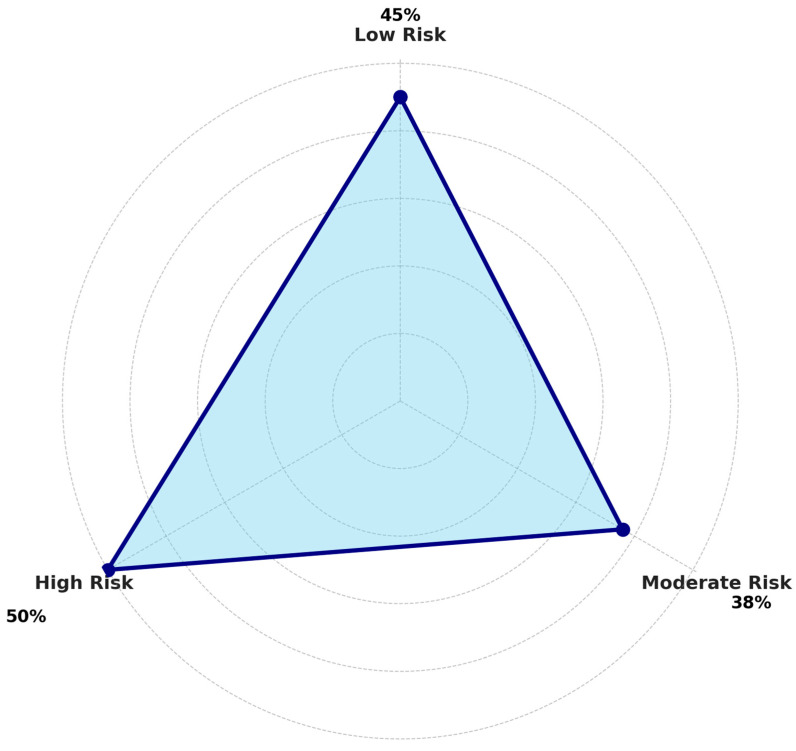
Proportion of Patients Diagnosed with DCM by Metabolic Risk Group. Radial plot showing the percentage of patients diagnosed with dilated cardiomyopathy (DCM) across metabolic risk groups. Risk categories were defined based on the cumulative presence of diabetes, thyroid dysfunction, and obesity. Percentages represent the prevalence of DCM within each group. Risk groups were defined by the cumulative presence of diabetes, obesity, and thyroid dysfunction (Low = 1 condition, Moderate = 2, High = 3). Low-risk group (n = 481), moderate-risk group (n = 240), and high-risk group (n = 89). Percentages represent prevalence rates within each group. Abbreviations and variables: Low Risk = one condition; Moderate Risk = two conditions; High Risk = all three (DM, obesity, and thyroid dysfunction).

**Figure 3:**
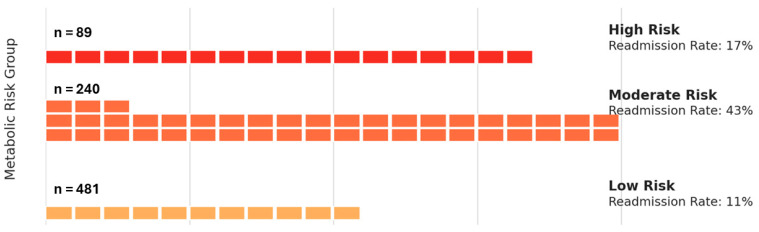
30-Day Hospital Readmission Rates by Metabolic Risk Group. Highlights clinical instability in moderate and high-risk patients. Abbreviations: DCM: Dilated Cardiomyopathy; DM: Diabetes Mellitus; Metabolic Risk Groups: Low Risk = one condition; Moderate Risk = two conditions; High Risk = all three (DM, obesity, and thyroid dysfunction).

The distribution of DCM and 30-day readmission rates across risk groups is visually depicted in Figure 2 and Figure 3, respectively, further illustrating the clinical gradient imposed by endocrine and metabolic burden. As illustrated in Figure 2, the prevalence of dilated cardiomyopathy (DCM) increased progressively across metabolic risk groups, from minimal to high risk. Patients with all three conditions—diabetes, obesity, and thyroid dysfunction—exhibited the highest DCM burden, suggesting a synergistic relationship between these endocrine/metabolic dysfunctions and structural cardiac pathology. Among the stratified metabolic risk groups, patients in the High-Risk category—defined by the coexistence of diabetes, obesity, and thyroid dysfunction—exhibited the highest prevalence of dilated cardiomyopathy (50%), consistent with a cumulative cardiometabolic burden effect. Interestingly, the Moderate Risk group (38%), characterized by the presence of two metabolic conditions, demonstrated a lower DCM prevalence than the Low Risk group (45%), which included patients with only one metabolic condition. This non-linear trend suggests potential heterogeneity within the Moderate Risk subgroup, possibly reflecting transitional disease states, earlier stages of structural cardiac remodeling, or selection effects related to treatment access, disease duration, or healthcare utilization. These findings underscore the need for more nuanced risk stratification models that account not only for the number but also the specific combinations and clinical context of metabolic comorbidities.

This radial (polar) plot displays the proportion of patients diagnosed with dilated cardiomyopathy (DCM) across three predefined metabolic risk groups: Low Risk: Patients with only one metabolic condition (e.g., diabetes, thyroid dysfunction, or obesity). Moderate Risk: Patients with two coexisting metabolic conditions. High Risk: Patients with all three conditions (diabetes, thyroid dysfunction, and obesity). Each axis segment represents one risk group. The distance from the center corresponds to the percentage of patients within that group who had DCM. The area under the curve is filled for visual emphasis. Data labels (e.g., “45%”) indicate the exact proportion per group. Risk categories were based on a composite Metabolic Burden Score, which accounts for the presence of diabetes mellitus, obesity, and thyroid dysfunction. DCM prevalence increases progressively from the minimal to the high-risk group.

Figure 3 shows that 30-day hospital readmission rates were disproportionately high in the moderate-risk group, surpassing even the high-risk category. This unexpected pattern may reflect complex interactions between partial endocrine burden and acute cardiovascular decompensation and emphasizes the need for close follow-up even in patients with intermediate risk profiles.

To visually compare short-term clinical instability across metabolic phenotypes, we constructed a waffle chart illustrating 30-day hospital readmission rates stratified by metabolic risk group. Risk categories were defined by the cumulative number of coexisting endocrine-metabolic conditions: Low Risk (one condition such as diabetes, obesity, or thyroid dysfunction), Moderate Risk (two conditions), and High Risk (all three). Each square (“brick”) in the chart represents approximately 1% of patients in that group who experienced a 30-day readmission. The number of filled squares reflects the observed readmission percentage: 11% for Low Risk, 43% for Moderate Risk, and 17% for High Risk. The horizontal axis denotes cumulative readmission percentage (0–100%), while the vertical grouping reflects risk stratification. This visual approach offers an intuitive comparison of post-discharge vulnerability across metabolic profiles, underscoring the disproportionately high early readmission rate among moderate-risk patients.

Waffle chart showing 30-day hospital readmission rates by metabolic risk group. Each square represents ~1% of patients readmitted within 30 days. Filled squares indicate the proportion of readmissions per risk group. Low-risk group (n = 481), moderate-risk group (n = 240), and high-risk group (n = 89). Percentages represent readmission rates within each group. Risk groups are defined by the number of metabolic conditions (diabetes, obesity, thyroid dysfunction): Low (1 condition), Moderate (2), High (3). Moderate-risk patients had the highest readmission rate (43%). Patients in the moderate-risk category exhibited the highest early readmission rates, suggesting that partial endocrine burden may significantly impact short-term clinical stability.

## 4. Discussion

This study investigated the independent and combined impact of endocrine-metabolic dysfunction—namely diabetes mellitus, thyroid disorders, and obesity—on the prevalence of dilated cardiomyopathy (DCM) and short-term clinical outcomes in a large real-world cohort. The principal finding is that non-insulin-treated diabetes and hypothyroidism are independently associated with a significantly increased risk of DCM, even after adjustment for age, sex, and other cardiovascular risk factors. Furthermore, a composite metabolic burden score reflecting the coexistence of diabetes, obesity, and thyroid dysfunction was associated with both a stepwise increase in DCM prevalence and a nonlinear pattern of 30-day hospital readmissions.

In our cohort of over 1000 patients, we observed that non-insulin-treated diabetes was more strongly associated with the prevalence of dilated cardiomyopathy (DCM) than either insulin-treated diabetes or obesity alone. This aligns with previous mechanistic models of diabetic cardiomyopathy that emphasize early cardiometabolic dysregulation—such as mitochondrial dysfunction, oxidative stress, advanced glycation end-products, and microvascular injury—as drivers of ventricular remodelling even before insulin dependency develops [16,17]. Existing reports underscore that diabetic cardiomyopathy can emerge in patients without overt macrovascular disease, consistent with our subgroup-based findings [18].

### 4.1. Non-Insulin-Treated Diabetes and Early Metabolic Injury

Our study provides strong evidence that non-insulin-treated diabetes and hypothyroidism are independent and potentially synergistic drivers of dilated cardiomyopathy (DCM), even after adjustment for age, sex, and cardiovascular comorbidities. Notably, non-insulin-treated diabetes was more strongly associated with DCM than either insulin-treated diabetes or obesity alone. This may reflect early cardiometabolic alterations—such as mitochondrial dysfunction, lipid toxicity, and microvascular injury—that promote myocardial remodelling before insulin dependence. These insights reinforce the evolving concept of diabetic cardiomyopathy as a distinct clinical entity, one that emerges independently of overt glycemic burden or macrovascular disease. While hypothyroidism and obesity have been previously identified as modifiers of heart failure progression, our findings are novel in demonstrating that their specific combination with non-insulin-treated diabetes confers the highest structural cardiac risk. Furthermore, our use of a composite Metabolic Burden Score provides a pragmatic framework for risk stratification and could serve as the basis for future predictive modelling tools aimed at identifying patients at risk of early structural heart disease [19,20].

Furthermore, the study cohort included 57.6% males and 42.4% females, indicating a moderate male predominance. While male sex was identified as an independent predictor of DCM in our multivariable models, this sex imbalance may influence the generalizability of our findings. The underrepresentation of female patients could be multifactorial ranging from lower rates of referral for cardiac imaging to differing symptom presentation to potential disparities in access to care in the study region. These factors raise important public health considerations, and future studies should explore whether sex-specific pathways or healthcare access inequities contribute to differential risk. Importantly, our exploratory sex-stratified analysis suggested consistent directionality of risk associations across sexes, but more adequately powered studies are needed to confirm these trends and investigate potential sex-specific modifiers of cardiomyopathy.

In our study cohort, males accounted for 57.6% of the population, indicating a moderate sex imbalance. While male sex emerged as an independent predictor of DCM in our multivariable analysis, this disproportion may influence the generalizability of our findings. Beyond biological susceptibility, the observed disparity may reflect systemic and societal factors such as reduced recognition of cardiac symptoms in women, differences in care-seeking behavior, and potential inequities in healthcare access within the study region. These considerations underscore the importance of incorporating sex- and gender-based analyses in cardiovascular research. Future prospective studies should evaluate whether these disparities affect diagnostic rates, disease severity, or treatment outcomes, particularly among women with metabolic comorbidities.

Additionally, the finding that non-insulin-treated diabetes was associated with a higher adjusted odds ratio for DCM than insulin-treated diabetes requires nuanced interpretation. While this may reflect genuine pathophysiological differences related to early metabolic dysfunction, confounding by disease duration or severity cannot be excluded. Patients on insulin may represent a later disease stage, with longer-standing diabetes, but also possibly better-recognized and managed cardiovascular risk. Conversely, those with non-insulin-treated diabetes may be under-monitored or under-treated, allowing silent cardiac remodelling to progress. Reverse causation is also a consideration, whereby patients developing DCM may have their diabetic management strategies modified post-diagnosis, thus influencing observed associations.

The Metabolic Burden Score further supports this interpretation, showing that DCM prevalence increases with cumulative metabolic dysfunction. Notably, non-insulin-treated diabetic patients with or without thyroid dysfunction exhibited the highest DCM rates, highlighting a critical window for early intervention. These patients may benefit from proactive cardiometabolic surveillance and early use of agents such as SGLT2 inhibitors, even before transitioning to insulin therapy [21].

Longitudinal and prospective cohort studies with systematic thyroid and metabolic biomarker assessments [22] will be essential to establish temporality and causal inference. Most prior high-impact studies rely on registry or epidemiologic data with limited clinical phenotyping, whereas ours is anchored in real-world EHR data with detailed endocrine segmentation.

### 4.2. Gender-Selective Risk

Our analysis revealed that male sex is independently associated with a more than twofold increase in the odds of developing DCM. This observation aligns with epidemiological data indicating higher DCM prevalence and earlier onset in men, potentially reflecting sex-related differences in myocardial remodelling, hormonal milieu, or genetic susceptibility. Notably, this sex-selective risk persisted even after adjusting for key metabolic exposures. These findings emphasize the need for sex-specific approaches in both risk stratification and preventive cardiology.

### 4.3. Thyroid Dysfunction and Cardiomyopathy

Subgroup analysis revealed heterogeneity in DCM prevalence across combinations of diabetes treatment and thyroid status. The highest risk was observed among non-insulin-treated diabetics, particularly those without thyroid dysfunction or with hypothyroidism. These patterns suggest additive or synergistic effects of metabolic derangement on cardiac structure. However, small subgroup sizes, especially in groups such as insulin-treated hyperthyroid patients, limit the reliability of some findings and require cautious interpretation. Further prospective studies are needed to confirm these phenotypic trends [23,24]. However, hyperthyroid patients constituted a smaller proportion of the cohort, and no cases of DCM were recorded among insulin-treated diabetic patients with hyperthyroidism. Although the subgroup was underpowered, the observed absence of DCM in insulin-treated hyperthyroid patients suggests potential endocrine interactions that merit further investigation in larger, prospective studies. A cohort study reported a moderate elevation of heart failure risk in older adults, even with subclinical hypothyroidism [25]. In heart failure populations, altered thyroid status has been associated with worse outcomes [14]. Our results reinforce these associations in a DCM-specific context, highlighting hypothyroidism as a potential independent driver of ventricular dilation.

By contrast, the absence of any DCM cases in hyperthyroid participants with insulin-treated diabetes—while intriguing—should be cautiously interpreted due to limited subgroup size. Previous literature does suggest that thyrotoxicosis may exacerbate heart failure via tachyarrhythmia and increased myocardial oxygen demand; yet it has not been systematically connected to a lower prevalence of DCM in diabetic subgroups [19]. The absence of DCM in insulin-treated hyperthyroid patients should be interpreted cautiously, given the small sample size (n = 54), which limits statistical power and generalizability.

### 4.4. Obesity: Independent Role and Interactions

Although obesity is widely recognized as a risk factor for heart failure, it was not independently associated with DCM in our multivariable models. This may reflect the mediating role of diabetes and thyroid dysfunction, or a dilution effect in a cohort where obesity prevalence was high (>70%). Nevertheless, obesity contributed meaningfully to the composite Metabolic Burden Score and influenced clinical outcomes when analysed in conjunction with other endocrine variables. The findings suggest that obesity alone may be insufficient to predict structural cardiac disease in this population, but that it amplifies risk in the presence of concurrent metabolic abnormalities. Obesity is a well-established independent risk factor in heart failure epidemiology [26,27], including obesity-related cardiomyopathy. Prior cohort data suggest that each unit increase in BMI significantly raises DCM risk [28]. However, in our multivariable models, obesity alone did not independently predict DCM after adjustment for diabetes and thyroid dysfunction. This finding may reflect obesity’s indirect role—acting through insulin resistance and hormonal dysregulation—rather than a direct structural effect. Indeed, autopsy and imaging studies have emphasized histopathological abnormalities in obesity-mediated cardiac remodelling that may require combined endocrine insults to manifest clinically [16].

The unexpectedly high 30-day readmission rate among moderate-risk patients suggests transitional clinical vulnerability despite partial metabolic burden. This group may represent individuals in the early stages of structural disease or those with under-recognized comorbidity clusters. Enhanced post-discharge monitoring in this intermediate-risk group may help reduce early clinical instability.

### 4.5. Subgroup Analysis: Endocrine Phenotypes and Clinical Outcomes

Subgroup analysis revealed heterogeneity in DCM prevalence across combinations of diabetes treatment and thyroid status. The highest risk was observed among non-insulin-treated diabetics, particularly those without thyroid dysfunction or with hypothyroidism. These patterns suggest additive or synergistic effects of metabolic derangement on cardiac structure. However, small subgroup sizes, especially in groups such as insulin-treated hyperthyroid patients, limit the reliability of some findings and require cautious interpretation. Further prospective studies are needed to confirm these phenotypic trends [29].

Importantly, the absence of DCM among insulin-treated hyperthyroid patients—while potentially suggestive—should not be overinterpreted. The small sample size (n = 54) substantially limits the statistical power of this subgroup, and the finding may represent a type II error or random variation rather than a true biological effect.

### 4.6. Comparison with Prior Literature

As described above, the early metabolic alterations in non-insulin-treated diabetes likely underlie the observed structural remodelling. Few previous studies have examined their combined influence on DCM using integrated statistical models and phenotypic subgrouping. While previous reports have identified hypothyroidism and obesity as modifiers of heart failure progression, our work provides novel evidence that specific combinations of endocrine dysfunction—notably non-insulin-treated diabetes and hypothyroidism—confer the highest structural cardiac risk. Moreover, the use of burden scores offers a practical framework for clinical risk stratification that could be adapted for future predictive modelling.

Numerous high-profile studies have examined the influence of metabolic dysfunction on cardiac structure and function, though often in isolation. Diabetic cardiomyopathy—characterized by ventricular dilation, interstitial fibrosis, metabolic dysregulation, and microvascular injury—is increasingly recognized as a distinct entity even without coronary artery disease or hypertension [30].

Our finding that non-insulin-treated diabetes is associated with the highest odds of dilated cardiomyopathy aligns with these mechanistic insights, suggesting that early metabolic changes—even before escalation to insulin therapy—may predispose to structural remodelling.

Obesity has long been implicated in adverse heart remodelling. A large Swedish cohort reported that each unit increase in BMI was associated with a 15% increased risk of DCM [31,32,33].

Yet, despite a high prevalence of obesity in our cohort, obesity alone did not independently predict DCM. This discrepancy may reflect differences in population characteristics or highlight that obesity’s impact is more pronounced when accompanied by endocrine dysfunction. Indeed, autopsy studies in Japan showed distinct morphological features in obesity cardiomyopathy, emphasizing that obesity alone is insufficient without histopathological assessment [34].

Our results, therefore, suggest that obesity magnifies risk primarily through its interaction with diabetes and thyroid dysfunction rather than acting as a standalone predictor.

Thyroid dysfunction plays a well-documented role in cardiovascular pathology, with hypothyroidism leading to impaired contractile function and diastolic dysfunction, and thyrotoxicosis sometimes precipitating cardiomyopathy in susceptible individuals [35].

Consistent with these data, hypothyroidism in our study was independently associated with increased DCM risk. Findings regarding hyperthyroidism were more nuanced: although no DCM cases were observed in the hyperthyroid subgroup with insulin-treated diabetes, this could reflect the small subgroup size. Other studies have demonstrated that thyrotoxicosis may accelerate heart failure progression and increase arrhythmic risk [36].

Recent reviews emphasize the concept of cardio-renal-metabolic multimorbidity in DCM, noting the high prevalence of metabolic syndrome, diabetes, and renal disease in patients with DCM and their compounded adverse impact on prognosis [26,37].

Our study supports this paradigm by demonstrating that composite endocrine burden scores correlate more closely with DCM and early clinical instability than isolated variables. This reinforces the notion that metabolic clustering, rather than single risk factor presence, drives disease progression.

### 4.7. Differences in Stratification and Modelling

Prior literature often treats diabetes, obesity, or thyroid disease as independent exposures, without systematically evaluating their joint effects. There is a dearth of clinical models that incorporate phenotypic subtyping, such as insulin treatment status or specific thyroid dysfunction categories. Our analytic approach, which used both ridge regression to reduce multicollinearity and subgroup phenotyping, diverges from traditional models and may explain why non-insulin-treated diabetes emerged with a stronger association than insulin-treated or obese phenotypes. This finding may reflect unique pathophysiologic features in early-stage metabolic disease, including lipid accumulation and inflammation, which precede insulin dependence [36].

### 4.8. Strengths over Prior Studies

In contrast to small single-center case series or autopsy studies of obesity-associated cardiomyopathy [38]. Our cohort includes over 1000 real-world patients with well-characterized endocrine and cardiac phenotypes. Importantly, the use of composite burden scores enabled us to stratify risk in a way that reflects clinical multimorbidity—a contrast to prior registry- or genetics-focused work that prioritized molecular aetiology or staging of DCM [39,40].

Compared with smaller studies or histopathologic investigations, our relatively large, phenotype cohort is a strength. The use of regularized regression to address multicollinearity among interrelated metabolic exposures also improves robustness.

However, limitations remain: our retrospective design and reliance on diagnosed thyroid and diabetes status—instead of standardized laboratory or biomarker thresholds—may underestimate subclinical disease prevalence. Prior prospective studies that used serial TSH, HbA1c, and imaging assessments have offered stronger causal insights but lacked subgroup-level analysis linking metabolic profiles to DCM.

### 4.9. Implications for Clinical Practice

These findings support a more nuanced and interdisciplinary approach to cardiovascular risk assessment. Routine cardiology evaluation of patients with diabetes and thyroid dysfunction may be warranted even in the absence of classic heart failure symptoms. Endocrinologists, cardiologists, and primary care providers should be aware of the compounded cardiac risk posed by coexisting metabolic disorders and consider joint screening strategies to identify patients at the highest risk of DCM and clinical decompensation. Incorporating composite metabolic indices into electronic health record alerts or care pathways could improve early detection and management of cardiomyopathy in high-risk populations. Incorporating the Metabolic Burden Score into EHR-based risk alerts may support earlier identification of DCM in high-risk endocrine profiles.

### 4.10. Limitations

This study is subject to several limitations that must be considered when interpreting the findings. First, the study cohort included a moderate predominance of male patients (57.6%), which may limit the generalizability of findings to the broader female population. Although sex was adjusted for in our multivariable models, underrepresentation of women may obscure sex-specific disease associations or risk modifiers. Second, the retrospective design inherently limits the ability to establish causal relationships. Although multivariable adjustment was performed to account for confounders, residual confounding from unmeasured clinical variables, such as medication adherence, socioeconomic status, or duration of endocrine disease, may still have influenced the results. Additionally, the reliance on existing electronic health records introduces potential documentation bias. Clinical diagnoses such as hypothyroidism or obesity may have been underreported or inconsistently coded, leading to potential misclassification of exposure variables. Moreover, detailed information on pharmacological therapy (e.g., antihypertensive drugs, oral antidiabetic agents, thyroid hormone replacement) and patient adherence was inconsistently documented in the retrospective dataset. As such, these factors could not be systematically included in the analysis. This limitation introduces the possibility of residual confounding, as variations in medication type, intensity, and compliance may have influenced the risk of DCM or readmission. Future prospective studies should incorporate standardized medication data and adherence measures to better account for these important modifiers of cardiometabolic risk.

Third, the heterogeneity in subgroup sample sizes, particularly in some endocrine phenotype combinations, reduced the statistical power to detect differences across all strata. For example, no cases of dilated cardiomyopathy were observed in the subgroup of insulin-treated patients with hyperthyroidism, but the small size of this group (n = 54) limits the generalizability and interpretability of this finding. Furthermore, the study lacked detailed data on the duration of endocrine conditions, particularly diabetes and thyroid dysfunction, which may significantly influence cardiovascular outcomes. Laboratory measures such as HbA1c for glycemic control and information on thyroid hormone replacement therapy were not systematically available and therefore could not be adjusted for. The absence of these variables may have introduced residual confounding and limited the ability to assess disease severity or treatment adequacy as modifiers of DCM risk. Similarly, stratified comparisons involving multiple combinations of thyroid and diabetes status may have been underpowered to detect nuanced interactions.

Fourth, the presence of perfect separation in some variable categories precluded the use of traditional logistic regression models, necessitating the use of regularized regression methods. While ridge regression allowed for robust model convergence and mitigated overfitting, it may introduce bias in the estimation of individual coefficients and complicate direct interpretation of marginal effects.

Fifth, the definitions of thyroid dysfunction and diabetes were based on documented clinical diagnoses rather than systematic laboratory measurements. This introduces the possibility that subclinical or undiagnosed cases may have been overlooked, particularly among euthyroid individuals or patients with impaired glucose tolerance who did not meet diagnostic thresholds for diabetes. Furthermore, the classification of thyroid status did not distinguish between treated and untreated disease, nor did it account for biochemical severity or duration of dysfunction, which may have important implications for cardiovascular risk. In particular, misclassification of thyroid status is a notable concern, as some patients had only limited or inconsistent thyroid function testing (e.g., missing free T4 or TSH outside of the index period), potentially leading to incorrect categorization as euthyroid or hypothyroid. This variability in testing may have attenuated or exaggerated the observed associations between thyroid dysfunction and DCM.

An important observation is that virtually all patients in our cohort had systemic hypertension, consistent with its high background prevalence in populations at risk of cardiomyopathy. While hypertension is well recognized as a contributor to adverse cardiac remodeling, its lack of statistical signal as an independent predictor in our models likely reflects this limited variability, rather than a true absence of association. Future prospective studies with more heterogenous cohorts are needed to disentangle the independent effects of hypertension from overlapping metabolic comorbidities.

Some subgroup analyses were underpowered due to small sample sizes. In particular, combinations such as insulin-treated diabetes with hyperthyroidism included fewer than 60 patients, limiting the ability to detect associations or generalize findings. The absence of observed events in these groups may reflect insufficient power rather than true clinical differences.

Finally, although the outcome of 30-day hospital readmission was clearly defined and validated, it encompassed all-cause admissions and was not restricted to cardiovascular events. While most readmissions were related to cardiac symptoms, the inclusion of non-cardiac causes may have diluted associations between metabolic risk and clinical instability. Moreover, the single-centre setting of the study may limit the external validity of the findings, as practice patterns and patient demographics may differ in other institutions or healthcare systems.

Subgroup sample sizes were sometimes small, reducing statistical power in stratified analyses. Additionally, perfect separation in some variables may have limited convergence in traditional regression models, necessitating regularization. Underpowered subgroup analyses may have produced misleading trends, particularly where no events were observed. Finally, causality cannot be inferred due to the observational nature of the data. Medication use (e.g., statins, thyroid hormone therapy) was not systematically captured or adjusted for, which may confound associations.

Taken together, these limitations underscore the need for future prospective studies using standardized diagnostic criteria, longer follow-up periods, and broader populations to confirm and extend the current findings. Despite these constraints, the study provides meaningful insights into the interdependence of endocrine and cardiovascular health and highlights key targets for risk stratification and preventive care in patients with metabolic disorders.

## 5. Conclusions

In this retrospective cohort study of over 1000 patients, endocrine-metabolic dysfunctions, particularly non-insulin-treated diabetes and hypothyroidism and male sex were independently associated with increased odds of DCM in this cohort. While the associations with non-insulin-treated diabetes, hypothyroidism, and male sex were consistent and statistically robust, subgroup trends (e.g., absence of DCM in insulin-treated hyperthyroid patients, nonlinear readmission rates across risk strata) should be interpreted as exploratory and hypothesis-generating due to limited sample size. These findings support the implementation of interdisciplinary screening and management approaches that integrate endocrine and cardiovascular care to slow the progression of heart failure. As a retrospective study, causality cannot be established, and prospective validation is required, as residual confounding cannot be excluded.

By critically contextualizing our results within existing high-impact literature, this study strengthens the evidence for non-insulin-treated diabetes and hypothyroidism as independent—and potentially synergistic—drivers of DCM. Moreover, it highlights obesity’s indirect role through its contribution to broader metabolic clustering. Collectively, our findings emphasize the importance of integrated cardiometabolic risk assessment and timely, targeted interventions aimed at preventing structural heart disease in metabolically vulnerable populations. Our results underscore the importance of integrating endocrine and sex-specific factors into DCM risk assessment. Prospective studies are warranted to validate these findings and to refine risk-stratification strategies that account for combined metabolic and demographic influences.

## Figures and Tables

**Table 1:** Baseline Clinical Characteristics of the Study Cohort.

Characteristic	n	%
Total Patients	1079	100
Gender (Male)	621	57.6
Insulin-treated diabetes	378	35
Non-insulin-treated diabetes	431	39.9
Diabetes not present	270	25
Obesity	756	70.1
Hypothyroidism	217	20.1
Hyperthyroidism	214	19.8
Hypertension	1079	100
Dilated Cardiomyopathy (DCM)	501	46.4

This table summarizes the baseline distribution of key metabolic, endocrine, and cardiovascular conditions among the 1079 patients included in the study. Patients were categorized based on diabetes treatment type, presence of obesity, thyroid dysfunction, hypertension, and diagnosis of dilated cardiomyopathy (DCM). These descriptive data provide context for subsequent analyses exploring the interrelation between metabolic-endocrine dysfunctions and cardiovascular outcomes. Abbreviations: DCM: Dilated Cardiomyopathy; n (%): Number of patients and percentage out of the total cohort.

**Table 2:** Top Independent Predictors of Dilated Cardiomyopathy (DCM) from Regularized Logistic Regression.

Predictor	Odds Ratio (OR)	95% CI	*p*-Value
Pulmonary hypertension (moderate)	0.05	0.01–0.31	0.002
Unknown hypertension status	0.60	0.28–1.29	0.19
Age (years)	0.99	0.97–1.01	0.23
Unknown obesity status	1.22	0.63–2.36	0.56
Euthyroid (no thyroid disorder)	1.30	0.72–2.36	0.38
Hypothyroidism	1.78	1.02–3.11	0.042
Male sex	2.33	1.36–4.00	0.002
Pulmonary hypertension (mild)	5.74	2.32–14.19	<0.001
Non-insulin-treated diabetes	6.93	3.78–12.73	<0.001
Diabetes not present	9.48	5.11–17.59	<0.001

Model Summary: AUC: 0.78 (95% CI: 0.74–0.82); accuracy: 74.6%; Hosmer–Lemeshow test: *p* = 0.23; Multicollinearity check: All VIFs < 2.5; no strong correlations (r < 0.40); Regularization penalty (lambda): Optimized via 10-fold cross-validation; AIC (non-regularized model): 1287.4 (for comparison). Abbreviations: DCM: Dilated Cardiomyopathy OR: Odds Ratio; Variable Descriptions: Age (years): Patient age at the time of admission; Male sex: Biological sex classified as male; Non-insulin-treated diabetes: Patients managed with oral antidiabetics and/or diet; Diabetes not present: Patients without a diabetes diagnosis; Hypothyroidism: Clinically documented thyroid hormone deficiency; Euthyroid (no thyroid disorder): Patients with normal thyroid function and no history of thyroid disease; Pulmonary hypertension (mild/moderate/severe): Physician-classified severity of pulmonary hypertension.

**Table 3:** Adjusted Predictors of Dilated Cardiomyopathy from Simplified Logistic Regression Model.

Predictor	Coefficient	Std. Error	Z-Value	*p*-Value	Adjusted Odds Ratio (95% CI)
Intercept	3.322	1.914	1.735	0.08	27.71
Diabetes not present (vs. insulin-treated)	−0.163	0.592	−0.276	0.78	0.85
Non-insulin-treated diabetes (vs. insulin-treated)	0.35	0.588	0.595	0.55	1.42
Hyperthyroidism (vs. euthyroid)	−0.875	0.606	−1.444	0.15	0.42
Hypothyroidism (vs. euthyroid)	−0.329	0.591	−0.558	0.57	0.72
Obesity (vs. not obese)	−0.341	0.477	−0.715	0.47	0.71
Age	−0.043	0.028	−1.505	0.13	0.96

This table summarizes the results of a simplified multivariable logistic regression model assessing the independent association between endocrine-metabolic variables and the risk of dilated cardiomyopathy (DCM). The model included diabetes status, thyroid function, obesity status, and age. The coefficients and corresponding odds ratios (ORs) reflect the adjusted effect of each predictor on the likelihood of DCM, with 95% confidence intervals (CI). The model was constructed to avoid convergence limitations observed in the full regression due to perfect separation. Abbreviations: OR—Odds Ratio; CI—Confidence Interval; Variable Descriptions: Diabetes not present (vs. insulin-treated): Patients without diagnosed diabetes compared to those treated with insulin; Non-insulin-treated diabetes (vs. insulin-treated): Patients using oral agents/diet only, relative to insulin users; Hyperthyroidism (vs. euthyroid): Overactive thyroid function versus normal thyroid status; Hypothyroidism (vs. euthyroid): Underactive thyroid function versus euthyroid state; Obesity (vs. not obese): Clinically diagnosed obesity compared to patients without obesity; Age (years): Continuous variable representing patient age at the time of inclusion.

**Table 4:** Clinical Outcomes by Metabolic Risk Stratification.

Risk Group	n	DCM Prevalence	30-Day Readmission Rate	Mean CV Burden Score	Mean Metabolic Burden Score
Low Risk	481	44.7	10.9	2.23	1
Moderate Risk	240	38.1	42.8	2.19	2
High Risk	89	50.2	16.7	2.33	3

DCM rate, 30-day readmission, and cardiovascular burden score across minimal, low, moderate, and high-risk groups. Risk groups were defined by the cumulative presence of diabetes, obesity, and thyroid dysfunction. Abbreviations: DCM: Dilated Cardiomyopathy; CV Burden Score: Cardiovascular Burden Score (sum of clinical CVD indicators including atrial fibrillation, n, sample size; NYHA class, HTA, and DCM); Metabolic Burden Score: Composite score (0–3) based on the presence of diabetes, thyroid dysfunction, and obesity.

## Data Availability

The data supporting the findings of this study are available from the corresponding authors upon reasonable request. Due to ethical and privacy considerations, the data are not publicly available.

## Abbreviations

The following abbreviations are used in this manuscript:

AUC	Area Under the Curve
BMI	Body Mass Index
CI	Confidence Interval
CVD	Cardiovascular Disease
DCM	Dilated Cardiomyopathy
DM	Diabetes Mellitus
EHR	Electronic Health Record
GCP	Good Clinical Practice
HTN	Hypertension
IRB	Institutional Review Board
NYHA	New York Heart Association
OR	Odds Ratio
SGLT2	Sodium–Glucose Cotransporter-2
T2DM	Type 2 Diabetes Mellitus
TSH	Thyroid-Stimulating Hormone
VIF	Variance Inflation Factor
